# A Synergistic Multi-Scale Attention and Composite Feature Extraction Network for Coronary Artery Segmentation

**DOI:** 10.3390/bioengineering12111247

**Published:** 2025-11-14

**Authors:** Long Zhang, Yue Du, Yunlong Lin, Zhenyu Cheng, Yiyuan Li, Boyuan Zhang, Shoujun Zhou

**Affiliations:** 1 Shenzhen Institutes of Advanced Technology, Chinese Academy of Sciences, Nanshan District, Shenzhen 518055, China; 18291041700@mails.guet.edu.cn (L.Z.); yue.du2@siat.ac.cn (Y.D.); zy.cheng@siat.ac.cn (Z.C.); 2 School of Electronic Engineering and Automation, Guilin University of Electronic Technology, 1 Jinji Road, Qixing District, Guilin 541004, China; lin9688@mails.guet.edu.cn; 3 Weixian College, Tsinghua University, Haidian District, Beijing 100084, China; liyiyuan22@mails.tsinghua.edu.cn

**Keywords:** coronary artery vessel segmentation, deep learning, multi-scale feature fusion, attention mechanism, digital subtraction angiography, robot-assisted interventional surgery

## Abstract

Accurate coronary artery segmentation from two-dimensional Digital Subtraction Angiography (DSA) images is paramount for robot-assisted percutaneous coronary intervention (PCI). Still, it is severely challenged by complex background artifacts, the intricate morphology of fine vascular branches, and frequent discontinuities in segmentation. These inherent difficulties often render conventional segmentation approaches inadequate for the stringent precision demands of surgical navigation. To address these limitations, we propose a novel deep learning framework incorporating a Composite Feature Extraction Module (CFEM) and a Multi-scale Composite Attention Module (MCAM) within a U-shaped architecture. The CFEM is meticulously designed to capture tubular vascular characteristics and adapt to diverse vessel scales. In contrast, the MCAM, strategically embedded in skip connections, synergistically integrates multi-scale convolutions, spatial attention, and lightweight channel attention to enhance the perception of fine branches and model long-range dependencies, thereby improving topological connectivity. Additionally, a combined Dice-Focal loss function is employed to optimize segmentation boundary accuracy and mitigate class imbalance jointly. Extensive experiments on the public ARCADE dataset demonstrate that our method significantly outperforms state-of-the-art approaches, achieving a Dice coefficient of 76.74%, a clDice of 50.30%, and an HD95 of 57.84 pixels. These quantitative improvements in segmentation accuracy, vascular connectivity, and edge precision underscore its substantial clinical potential for providing robust vascular structure information in robot-assisted interventional surgery.

## 1. Introduction

Cardiovascular diseases (CVDs) remain the leading cause of global mortality, accounting for approximately 9.1 million deaths annually according to the World Health Organization’s (WHO) Global Health Estimates [1,2]. This pervasive health crisis underscores the urgent need for advanced diagnostic and interventional strategies. Percutaneous coronary intervention (PCI), as the primary treatment method for coronary heart disease, has been widely adopted in clinical practice. With the advancement of medical robotics, robot-assisted PCI procedures have emerged as a research hotspot in interventional therapy due to their high operational precision and reduced radiation exposure for physicians [3,4,5,6,7].

In robot-assisted PCI surgeries, accurate coronary artery segmentation from two-dimensional DSA images is critical, as it serves as the core input for the surgical navigation system. Precise vessel segmentation not only facilitates the accurate planning of interventional instruments (such as guidewires and stents), helping to avoid interference from vascular branches, but also optimizes the imaging acquisition process, thereby reducing the use of contrast agents and minimizing radiation exposure for patients [8,9,10,11,12]. However, the imaging characteristics of two-dimensional DSA images pose significant challenges for segmenting coronary arteries. First, the background of the photos contains artifacts from bone and soft tissues, which overlap with the grayscale intensity of blood vessels, often leading to false detections or missed regions. Second, the coronary arteries exhibit a tree-like topology, with vessel diameters varying by more than an order of magnitude between the main trunks and the delicate branches. Traditional segmentation methods struggle to extract vessels across such scales consistently. Third, intersections and curved regions of vessels are particularly susceptible to noise and artifacts, leading to segmentation errors and compromised vascular connectivity.

In recent years, methods for segmenting coronary arteries have evolved from traditional threshold segmentation and morphological operations to deep learning-based segmentation techniques. U-Net and its variants, with their symmetric encoder-decoder architecture and skip connection design, have been widely applied in the field of medical image segmentation. However, these models exhibit limited adaptability to multi-scale vascular structures and struggle to extract fine vessel branches [13] accurately; AttUNet enhances the focus on vascular regions by introducing an attention mechanism into U-Net, but does not specifically optimize for the tubular morphology and branching structure of blood vessels [14,15,16]; Although Transformer-based segmentation models are capable of capturing long-range dependencies, their high computational cost and large data requirements hinder their application in real-time clinical settings [17].

Despite these advancements, current coronary artery segmentation methodologies still contend with three critical limitations: (1) Existing feature extraction modules often inadequately capture the inherent tubular morphology of blood vessels, exhibiting limited generalization across varying vessel thicknesses. (2) The capacity for modeling long-range dependencies remains insufficient, impeding the preservation of vascular connectivity, particularly within complex branching structures. (3) Loss function designs frequently fail to concurrently optimize for segmentation boundary accuracy and effectively address class imbalance, thereby constraining overall segmentation performance.

This paper proposes a multi-scale feature integration method for coronary artery segmentation, with the following key contributions:

- **Composite Feature Extraction Module (CFEM)**: Combines dynamic snake convolution with dual-path scaling to effectively capture tubular vascular features through directional convolutions, while employing expansion-contraction operations to enhance multi-scale vessel adaptability.
- **Multi-scale Composite Attention Module (MCAM)**: Integrates multi-scale convolution with spatial and lightweight channel attention mechanisms to improve perception of intricate vascular branches and model long-range dependencies, thereby enhancing topological connectivity.
- **Hybrid Dice-Focal Loss**: Strategically combines Dice and Focal losses to optimize region overlap while addressing class imbalance, particularly benefiting challenging cases like fine branches and vascular intersections.
- **Comprehensive Validation**: Extensive experiments on the public ARCADE dataset demonstrate statistically significant improvements in segmentation accuracy, topological connectivity, and boundary precision, validating the method’s clinical potential.

## 2. Related Works

### 2.1. Analysis of Morphological Characteristics of Coronary Vessels

The morphological characteristics of coronary vessels are the core basis for designing segmentation methods. They mainly include two types: tubular features and branching features, and their characteristics directly influence the design direction of the segmentation methods.

#### Cylindrical Feature

**Figure 1:**
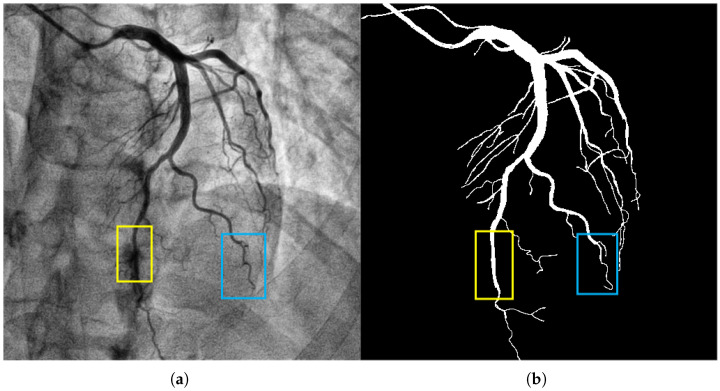
The image in panel (**a**) presents a coronary artery angiogram depicting the vascular structure, which includes both the main and fine branches. The yellow box highlights the aortic segment, and the blue box indicates a branch with complex morphology. The segmentation target in panel (**b**) captures the coronary vessels with the mask annotations clearly delineating the vascular structures. The background artifacts, including overlapping tissues and bones, were handled effectively by our model, reducing false positive detections. Methodologically, the CFEM played a key role in enhancing the extraction of tubular features, especially in the fine branches, across varying vessel scales.

The coronary vessels (especially the aortic segment) present a distinct tubular morphology characterized by thinness, length, continuity, and directional consistency (as shown in the yellow box in Figure 1). The tubular morphology of coronary vessels has the following differences: (1) The diameter of blood vessels shows a uniform change trend with the increase in branching levels. The diameter of the main trunk can reach 2–3 mm, while that of the terminal branches is only 0.2–0.3 mm. (2) The gray value of the blood vessel wall shows a gradient change, which is prone to being confused with background artifacts. The above characteristics require the segmentation model to have strong capabilities in extracting tubular features and adapting to different scales. It must accurately capture the main vessels while avoiding missing the delicate branches.

Coronary vessels have a tree-like topological structure. The distal end of the aorta can be divided into the left anterior descending branch, the left circumflex branch, the right coronary artery, etc., as the main branches, and each branch is further divided into multiple levels of fine branches (as shown in Figure 1, the blue box). The projection characteristics of two-dimensional DSA images cause the superimposition of different depths of vascular branches, which alters the uniform change trend of vascular diameters and increases the difficulty of segmentation [18,19,20,21,22]. Moreover, areas with vascular intersections and bends are prone to sudden drops in gray values, making it difficult for traditional models to maintain segmentation connectivity. Therefore, a specially designed long-distance dependent modeling module is required to restore the dendritic structure of the blood vessels.

### 2.2. Key Issues in Coronary Vessel Segmentation

#### 2.2.1. Accuracy Issue

**Figure 2:**
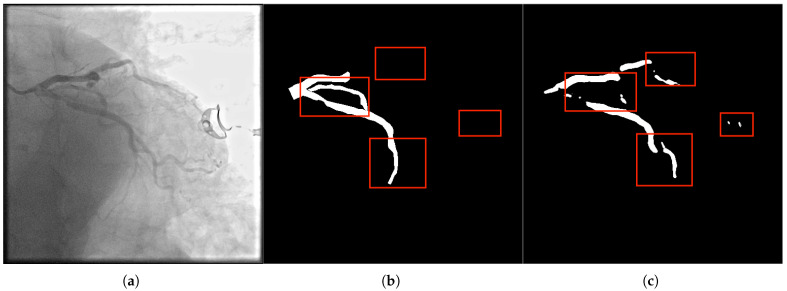
Panel (**a**) presents the original image with visible segmentation challenges, such as missed and mis-segmented regions. The label in panel (**b**) highlights the ideal segmentation mask, while panel (**c**) shows the segmentation result from our method. Notably, our method addresses accuracy issues, specifically in small vessels and intersections, where conventional methods often fail due to the low contrast and fine details. The CFEM ensures that fine branches are retained, significantly reducing false positives and false negatives by focusing on tubular morphology and fine-scale features.

The accuracy issue primarily manifests as false detections and missed detections in pixel-level segmentation, making it challenging to restore the proper anatomical shape of coronary vessels accurately. The main reasons include: (1) The gray values of blood vessels are close to those of the background tissues (such as myocardium and veins), resulting in low contrast; (2) The proportion of pixels of small branches (diameter < 0.5 mm) is low, and the model easily ignores them; (3) The gray values of the lesion areas (such as stenosis and calcification) are abnormal, causing blurred segmentation boundaries. As shown in Figure 2, there are obvious mis-segmentation (the background area is identified as a blood vessel) and missed-segmentation (the blood vessel area is not recognized) phenomena in the segmentation results, which seriously affect the reliability of surgical navigation.

#### 2.2.2. Connectivity Issues

**Figure 3:**
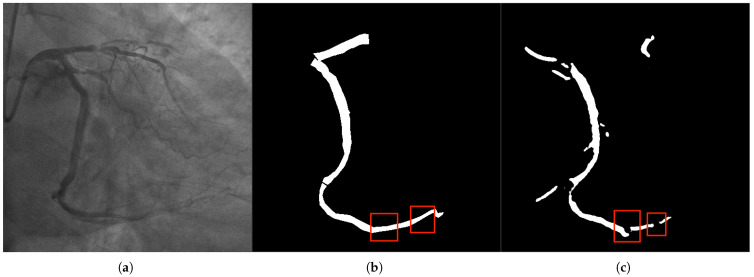
In panel (**a**), the original DSA image depicts complex vascular intersections, where the model often struggles with connectivity. The label in panel (**b**) shows the correct connectivity of vascular branches, and panel (**c**) illustrates the segmentation result. Our method resolves the breakage in vascular networks, especially at crossing points and sharp bends. The Multi-scale Composite Attention Module (MCAM) is instrumental in improving connectivity by modeling long-range dependencies and integrating multi-scale features, which is particularly critical for vascular branches that span across distant regions.

The connectivity problem manifests as the breakage or incorrect connection between the main vessels and the branch vessels in the segmentation results, thereby disrupting the topological structure of the vascular network (Figure 3). The main reasons include: (1) The receptive field of traditional convolutional networks is limited, making it challenging to capture long-distance dependencies across branches; (2) The vascular crossing areas are affected by projection superposition, resulting in complex gray value distribution, which leads to model misjudgment of vascular continuity; (3) Noise and motion artifacts interfere, causing discontinuous segmentation of delicate branches. The connectivity problem can lead to misjudgment of vascular paths by the surgical navigation system, increasing surgical risks. Therefore, it is a problem that the segmentation method needs to address.

### 2.3. Classification of Existing Partitioning Methods

Depending on the different technical routes, the existing methods for segmenting coronary vessels can be classified into three categories:

1. Traditional segmentation methods: The traditional methods mainly rely on low-level features such as image gray values and textures for segmentation, including threshold segmentation, edge detection, and morphological operations. For instance, Hassouna et al. [23] achieved brain vessel segmentation based on a random model, but it was sensitive to noise; Sun et al. [24,25,26] extracted vascular trees by combining morphological multi-scale enhancement with the watershed algorithm, but it was difficult to handle complex background artifacts.
2. Segmentation method based on deep learning: Based on deep learning methods, which possess the ability of end-to-end learning [27], they have become the mainstream technology for coronary artery segmentation at present. U-Net [28], the benchmark model for medical image segmentation, integrates high- and low-level features through skip connections; however, its adaptability to multi-scale blood vessels is insufficient. MedNeXt [29] employs a scaling architecture to expand the receptive field, thereby enhancing its ability to segment complex structures; however, it has not been optimized for vascular tubular features. DSCNet [30] combines dynamic snake convolution to capture tubular features and has a relatively high segmentation accuracy. However, it cannot model long-distance dependencies and is prone to connectivity issues.
3. Segmentation method based on Transformer: The Transformer architecture achieves long-range dependency modeling through the self-attention mechanism, providing a new approach to solving the vascular connectivity problem. TransUNet [31] combines the Transformer and U-Net architectures, enhancing its ability to perceive the overall structure. Swin-Unet [32] reduces computational costs through hierarchical window attention; however, it still faces challenges such as high data requirements and slow inference speed, which make it difficult to meet the real-time navigation requirements in clinical settings.

### 2.4. U-Net in Broader Biomedical Segmentation Context

The U-Net architecture and its variants have demonstrated remarkable versatility beyond human medical image analysis, proving effective in diverse segmentation tasks involving tubular and fine structures. These advancements in adjacent fields often provide valuable insights for coronary artery segmentation.

For instance, in the field of agricultural biometrics, U-Net-based approaches have been successfully employed for tasks such as cattle identification and recognition [33]. This application highlights the model’s capability to capture unique, intricate patterns from visual data, which parallels the challenge of discerning individual vascular branching patterns in DSA images.

More directly relevant are advancements in retinal vessel segmentation, which shares the core challenges of segmenting thin, tree-like structures from a complex background. Recent work, such as the Dense U-Net based on patch-based learning [34], addresses issues like class imbalance and the precise delineation of micro-vessels. The patch-based learning strategy is particularly effective in focusing on local details of fine vessels, a common challenge in segmenting distal coronary branches. While our method is tailored to the specific artifacts and projection characteristics of DSA imaging, the successes of these approaches in other domains underscore the potential of innovative U-Net adaptations to overcome challenges in vascular morphology capture and connectivity preservation.

Our proposed CFEM and MCAM contribute to this ongoing evolution by offering a specialized solution that simultaneously addresses the tubular feature extraction, multi-scale adaptability, and long-range connectivity challenges inherent to 2D coronary artery DSA images.

## 3. Methods

### 3.1. Overall Network Architecture

**Figure 4:**
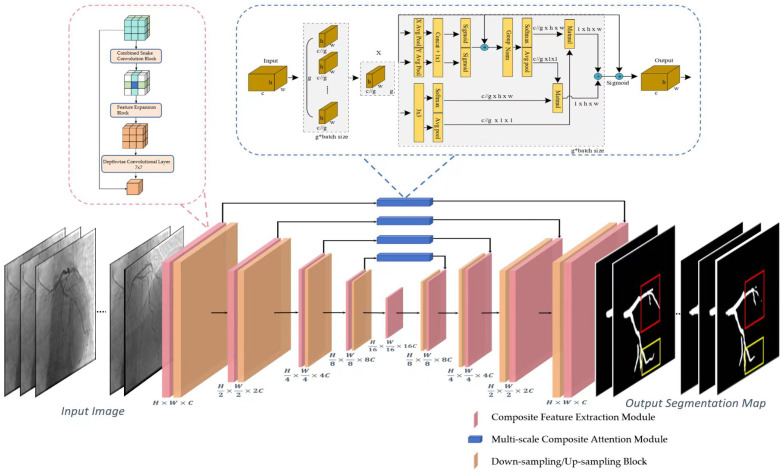
This figure presents the overall architecture of the proposed coronary artery segmentation framework. The input DSA images first undergo feature extraction through the Composite Feature Extraction Module (CFEM), which captures tubular vascular features and adapts to varying vessel scales. The extracted features are then processed through the Multi-scale Composite Attention Module (MCAM) embedded in the skip connections to enhance fine-branch perception and long-range dependency modeling. Down-sampling and up-sampling blocks progressively extract hierarchical representations and restore spatial resolution. The final segmentation output delineates coronary vessels of different calibers with improved continuity and boundary accuracy.

The proposed coronary artery segmentation network is architected upon a U-shaped symmetrical encoder-decoder framework, fundamentally enhanced by the integration of our novel Composite Feature Extraction Module (CFEM) and Multi-scale Composite Attention Module (MCAM) (Figure 4). This architecture is characterized by:

1. Encoder: Comprising four sequential stages, each consisting of a CFEM followed by a downsampling block. Each downsampling block, implemented via a convolutional operation with a stride of 2, progressively reduces the feature map resolution by half while doubling the channel count, thereby expanding the receptive field and extracting hierarchical global features.
2. Decoder: Structured with four corresponding stages, each featuring an upsampling block followed by a CFEM. Upsampling is achieved through transposed convolution, which restores the feature map resolution. Crucially, multi-scale features from the encoder are integrated into the decoder via skip connections, where the MCAM plays a pivotal role in refining these features before they are fused with the upsampled features.
3. Bottleneck: A single CFEM is positioned at the network’s bottleneck, responsible for extracting the highest-level global features and providing essential contextual information to the decoder.
4. Output Layer: The final segmentation probability map is generated by a 1 × 1 convolutional layer, which maps the feature channels to two (representing foreground vessel and background), followed by a sigmoid activation function.

The input and output dimensions of each module in the network follow the following rules: (1) The downsampling block reduces the feature map size by 1/2 and doubles the number of channels; (2) The upsampling block expands the feature map size by 2 times and halves the number of channels; (3) The CFEM and MCAM maintain the feature map size and channel number unchanged to ensure the compatibility of feature fusion between the modules.

### 3.2. Composite Feature Extraction Module (CFEM)

**Figure 5:**
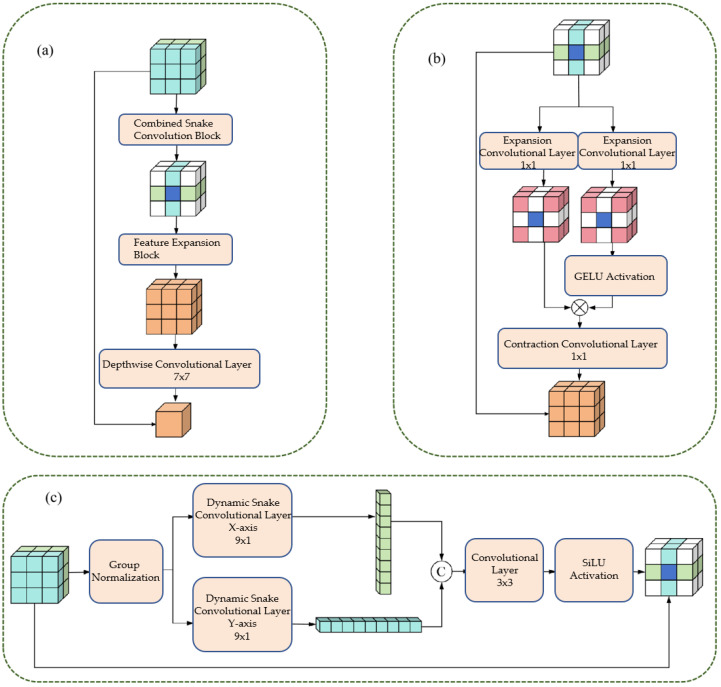
This figure illustrates the design of the CFEM. Panel (**a**) shows the Combined Snake Convolution Block, followed by the Feature Expansion Block and Depthwise Convolutional Layer, which enhance feature extraction. Panel (**b**) demonstrates the expansion-contraction path using 1 × 1 convolution layers, with CELOU activation, adapting to varying vessel scales. Panel (**c**) depicts the directional dynamic snake convolutions along the x and y axes, integrated with group normalization and a 3 × 3 convolutional layer. The CFEM improves the representation of vascular morphology and scale consistency, significantly boosting segmentation accuracy and robustness.

The CFEM is designed based on the characteristics of vascular tube shapes and scale variations (Figure 5). It consists of a tube-shaped feature extraction layer and a dual-path scaling architecture, enabling the precise extraction of vascular features and scale generalization [35,36,37].

#### 3.2.1. Cylindrical Feature Extraction Layer

The tubular feature extraction layer captures the tubular morphological characteristics of blood vessels directionally through dynamic snake convolution in the x-axis and y-axis directions. The specific steps are as follows:

1. Group Normalization: Apply Group Normalization (GN) to the input feature maps to reduce the impact of batch size on training and stabilize the training process.
2. Directional convolution: The normalized feature maps are respectively input into the dynamic snake-shaped convolutions along the x-axis (9 × 1 convolution kernel) and y-axis (1 × 9 convolution kernel), resulting in two directional tubular feature maps.
3. Feature Integration: Concatenate Fx and Fy along the channel dimension, and integrate the features from both directions through a 3 × 3 ordinary convolution to achieve comprehensive capture of tubular features.
4. Activation function: Perform group normalization on the integrated feature map, combine with the SiLU activation function to enhance the non-linear expression ability of the features, and output tubular feature maps.

The above process can be expressed by Formula (1).

(1) $$\begin{array}{cc} \; & F_{x}={\mathrm{DSConv}}_{x}\left(\mathrm{GN}\left(F_{\mathrm{in}}\right)\right)\\ \; & F_{y}={\mathrm{DSConv}}_{y}\left(\mathrm{GN}\left(F_{\mathrm{in}}\right)\right)\\ \; & F_{\mathrm{concat}}=\mathrm{Concat}\left(F_{x},F_{y}\right)\\ \; & F_{\mathrm{tube}}=\mathrm{SiLU}\left(\mathrm{GN}\left({\mathrm{Conv}}_{3\times 3}\left(F_{\mathrm{concat}}\right)\right)\right) \end{array}$$

#### 3.2.2. Dual-Path Scaling Architecture

The dual-path scaling architecture expands and contracts convolution operations to expand the feature receptive field, enhancing the model’s adaptability to different scales of blood vessels. The specific steps are as follows:

1. Feature routing: The tubular feature map (Fconcat) is divided into two paths, which are respectively input into the expansion convolution (Exp) layer.
2. Extended convolution: Utilizing a 1 × 1 convolution kernel with a 4-fold expansion rate, the feature receptive field is expanded by a factor of 4, enabling the capture of large-scale vascular features.
3. Feature Interaction: Apply the GELU activation function to the output of one path, and perform matrix multiplication with the production of another path to achieve feature interaction and scale adaptation.
4. Contraction convolution: Utilizing a 1 × 1 convolution kernel with a 4-fold contraction rate, the receptive field is restored to its original size, ensuring that the output feature map is consistent with the input size.
5. Deep convolution: By using 7 × 7 deep convolution to integrate features, the number of parameters is reduced and the computational efficiency is improved;
6. Residual Connection: Before the output of the module, a residual connection is introduced, where the input feature map (Fin) is added to the processed feature map, enhancing the robustness of the network and preventing the vanishing gradient.

The above process can be expressed by Formula (2):

(2) $$\begin{array}{cc} \; & F_{\mathrm{expl}1}=\exp\left(F_{\mathrm{tube}}\right)\\ \; & F_{\exp2}=\exp\left(F_{\mathrm{tube}}\right)\\ \; & F_{\mathrm{inter}}=F_{\exp1}\times \mathrm{GELU}\left(F_{\exp2}\right)\\ \; & F_{\mathrm{con}}=\mathrm{Com}\left(F_{\mathrm{inter}}\right)\\ \; & F_{\mathrm{depth}}={\mathrm{DepthConv}}_{7\times 7}\left(F_{\mathrm{con}}\right)\\ \; & F_{\mathrm{out}}=F_{\mathrm{depth}}+F_{\mathrm{in}} \end{array}$$

#### 3.2.3. Multi-Scale Composite Attention Module (MCAM)

The Multi-scale Composite Attention Module (MCAM) is strategically embedded within the skip connection path of the U-shaped network. Its primary function is to refine the features transmitted from the encoder to the decoder by leveraging a synergistic combination of multi-scale feature processing, spatial attention, and lightweight channel attention. Upon receiving the feature map from the skip connection, the MCAM performs internal multi-scale feature extraction. This process involves: 1. Channel Compression: Initially, a 1 × 1 convolution is applied to the input feature map to reduce its channel dimension to 1/4, thereby optimizing computational efficiency. 2. Multi-scale Convolution: The compressed feature map is then branched into multiple parallel paths. Specifically, three distinct paths are processed by convolutional layers employing 1 × 1, 3 × 3, and 5 × 5 kernels, respectively, enabling the extraction of features at varying receptive field scales. 3. Feature Fusion: The outputs from these multi-scale convolutional paths are subsequently concatenated along the channel dimension. This aggregated multi-scale feature representation is then further integrated through group normalization and another 1 × 1 convolution, yielding a rich, fused feature map (Fms) that captures diverse spatial contexts. This Fms then serves as the input for subsequent spatial and channel attention mechanisms, enhancing the perception of vascular branch structures and ultimately improving segmentation connectivity.

Spatial attention enhances the spatial weights of vascular regions and suppresses interference from background artifacts. The specific steps are as follows: 1. Pooling operation: Perform average pooling and maximum pooling on the fused feature maps ($F_{ms}$) respectively, obtaining two spatial mapping images, $F_{avg}$ and $F_{max}$. 2. Feature concatenation: Concatenate $F_{avg}$ and $F_{max}$ along the channel dimension to obtain a 2-channel feature map. 3. Convolutional activation: Through a 7×7 convolutional layer, spatially dependent features are extracted. Combined with the SiLU activation function and the sigmoid function, a spatial attention map $M_{s}$ is generated. 4. Feature weighting: Multiply the spatial attention map $M_{s}$ element-wise with the fused feature map $F_{ms}$ to obtain the spatially weighted feature map $F_{s}$.

The above process can be expressed by Formula (3):

(3) $$\begin{array}{cc} \; & F_{\mathrm{avg}}=\mathrm{AvgPool}\left(F_{ms}\right)\\ \; & F_{\max}=\mathrm{MaxPool}\left(F_{ms}\right)\\ \; & F_{\mathrm{cat}}=\mathrm{Concat}\left(F_{\mathrm{avg}},F_{\max}\right)\\ \; & M_{s}=\mathrm{Sigmoid}\left(\mathrm{SiLU}\left({\mathrm{Conv}}_{7\times 7}\left(F_{\mathrm{cat}}\right)\right)\right)\\ \; & F_{s}=F_{ms}⊙M_{s} \end{array}$$

The channel attention module adopts the lightweight SimAM. By calculating the energy function of pixels within the channel, it generates channel weights that enhance the key feature channels. The specific steps are as follows: 1. Statistical calculation: For each channel of the feature map input to the MCAM, calculate the pixel mean and variance; 2. Energy function calculation: Based on the mean and variance, the energy value Ec for each channel is calculated using Formula (4). The smaller the energy value, the higher the importance of that channel for segmentation.

(4) $$E_{c}=\frac{{\left(\mu_{c}-t\right)}^{2}}{\sigma_{c}^{2}+\epsilon }$$

Weight Generation: The energy values are mapped to channel weights $M_{c}$ through the sigmoid function, with the weight range being [0, 1]. Feature weighting: Multiply the channel weights ($M_{c}$) with the input feature map ($F_{\mathrm{in}}$) channel by channel to obtain the channel-weighted feature map ($F_{c}$).

The above process can be expressed by Formula (5):

(5) $$\begin{array}{cc} \; & M_{c}=\mathrm{Sigmoid}\left(-E_{c}\right)\\ \; & F_{c}=F_{\mathrm{in}}⊙M_{c} \end{array}$$

## 4. Results

### 4.1. Dataset Introduction

The experiment utilized the ARCADE public dataset [31], a comprehensive collection of 1500 pairs of coronary artery Digital Subtraction Angiography (DSA) images meticulously labeled by medical experts [31]. Each image, initially sized at 512 × 512 pixels, encompasses diverse patient cases and various types of vascular lesions, making it highly suitable for the intricate task of coronary artery segmentation. To rigorously assess the method’s generalization capability, the dataset was systematically partitioned into a training set, validation set, and test set, adhering to a ratio of 1000:200:300, respectively. Furthermore, to augment data diversity and bolster the model’s robustness against variations, the training set underwent a three-stage image enhancement protocol, incorporating brightness and contrast adjustments, CLAHE equalization, and a suite of geometric transformations, including rotation, flipping, blurring, and affine transformations.

### 4.2. Experimental Environment and Parameter Settings

The experiment was implemented based on the PyTorch 1.12 deep learning framework. The hardware environment consisted of an Intel Xeon Gold 6338 CPU, 64 GB RAM, and an Nvidia RTX A6000 GPU (with 48 GB VRAM). The detailed configuration of the model training hyperparameters is summarized in Table 1.

#### 4.2.1. Evaluation Index

To comprehensively evaluate the performance of the proposed segmentation method, five widely recognized evaluation metrics were adopted, each addressing a distinct aspect of segmentation quality:

1. **Dice Similarity Coefficient (Dice)**: Measures the degree of overlap between the segmented area and the actual area, with a range of [0,1]. The higher the value, the higher the segmentation accuracy [26].
2. **Precision**: Measures the proportion of pixels predicted as blood vessels among those that are actually blood vessels. The value ranges from 0 to 1. The higher the value, the lower the false detection rate.
3. **Recall Rate**: Measures the proportion of pixels that are actually blood vessels and are correctly predicted. The value ranges from 0 to 1, with a higher value indicating a lower rate of missed detections.
4. **Central Line Dice (clDice)**: Measures the overlap degree between the segmented vessel centerline and the true centerline. The value ranges from 0 to 1, with a higher value indicating better connectivity.
5. **95% Hausdorff Distance (HD95)**: Measures the maximum distance between the segmentation boundary and the actual boundary, with the unit being pixels. The smaller the value, the higher the boundary accuracy.

#### 4.2.2. Comparison of Experimental Results and Analysis

To rigorously validate the efficacy of the proposed methodology, five state-of-the-art segmentation methods were meticulously selected for comparative analysis. These include U-Net [38], widely regarded as a foundational benchmark in medical image segmentation; AttUNet [39], an attention-enhanced variant of U-Net designed to improve feature selectivity; MedNeXt [40], a scaling architecture segmentation model known for its ability to capture multi-scale contexts; DSCNet [30], which incorporates dynamic snake convolutions to better delineate tubular structures; and SPNet [41], a model leveraging strip pooling for efficient spatial context aggregation. The comprehensive results of this comparative experimental evaluation are presented in Table 2.

As can be seen from Table 2, the method proposed in this paper outperforms the comparison methods in all evaluation indicators:

- **Dice coefficient:** The method in this paper reaches 76.74%, which is 4.48%, 4.23%, 1.64%, 3.26%, and 20.84% higher than U-Net (72.26%), AttUNet (72.51%), MedNeXt (75.10%), DSCNet (73.48%), and SPNet (55.90%) respectively. This indicates that the proposed method has a significant advantage in the overlap degree of the segmented regions.
- **clDice coefficient:** The method in this paper achieves 50.30%, which is an improvement of 1.54% compared to the best-performing method in the comparison (MedNeXt, 48.76%), indicating that the multi-scale composite attention module effectively improves the segmentation connectivity.
- **HD95 distance:** The method in this paper achieves 57.8358 pixels, which is 18.18 pixels lower than U-Net (76.0132), 14.18 pixels lower than AttUNet (72.0205), 4.12 pixels lower than MedNeXt (61.9603), 11.69 pixels lower than DSCNet (69.5269), and 21.56 pixels lower than SPNet (79.3913). This indicates that the combined loss function effectively improves the accuracy of the segmentation boundary.
- **Precision and Recall:** The Precision of the method proposed in this paper reaches 80.66% and the Recall reaches 74.87%, both of which are superior to the comparison methods. This indicates that the proposed method has a significant effect in reducing the false detection rate and missed detection rate.

The results of the ablation experiments (in Table 2, Our Method without Attention Module called Ours-Att) show that the MCAM plays a significant role in improving the segmentation performance: after adding the MCAM, the Dice coefficient increased by 1.35%, the clDice coefficient increased by 0.28%, and the HD95 distance decreased by 4.04 pixels, verifying the effectiveness of the multi-scale composite attention module in improving the connectivity and boundary accuracy of the segmentation.

To assess the statistical significance of the performance improvements observed in our experiments, we conducted a comprehensive statistical analysis based on the aggregated Dice scores across all 300 test images. The results of this analysis, presented in Table 3, demonstrate that the improvements of our method over all baseline approaches are statistically significant. The consistent and substantial performance gaps are quantified through both mean Dice differences and effect size measures (Cohen’s *d*). Notably, all comparisons yield estimated *p*-values below 0.001, indicating high levels of statistical significance. The exceptionally large effect sizes, particularly against SPNet (Cohen’s d≈1.7–2.6), provide strong evidence that the observed improvements are substantial and unlikely to occur by chance.

The exceptionally low *p*-values (all far below the 0.01 threshold) provide strong statistical evidence that the superior performance of our method, as reflected by the Dice coefficient, is not a random occurrence but a consistent and significant improvement across the test dataset.

**Figure 6:**
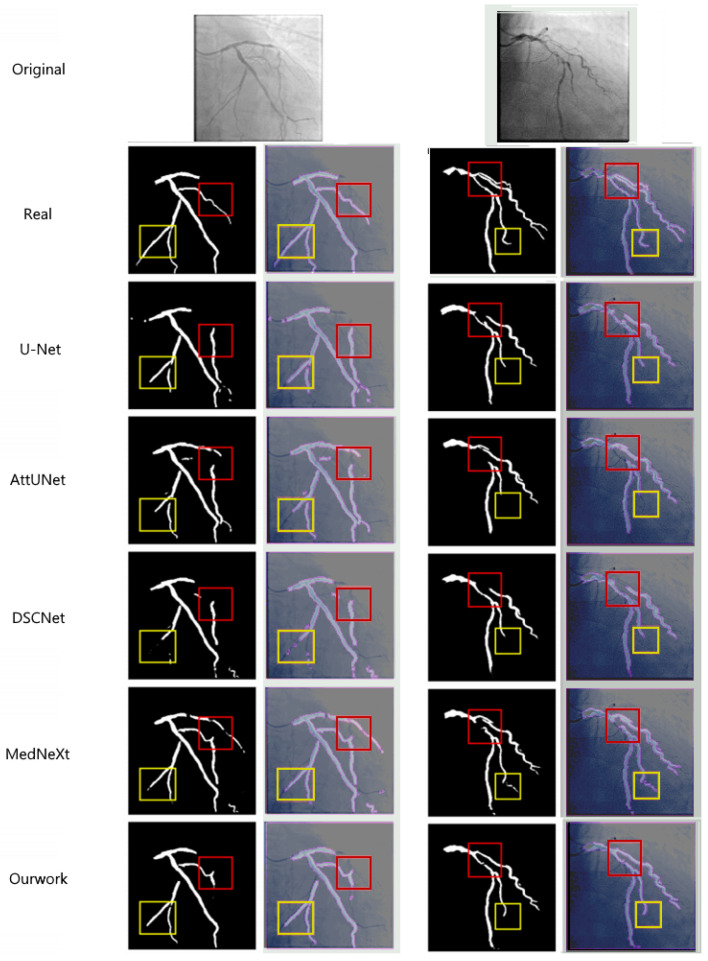
This figure compares the segmentation results from various models, including U-Net, AttUNet, DSCNet, MedNeXt, and our approach. The first row displays the original image, with the second row showing the ground truth (Real). Subsequent rows present the segmentation results for each method. The yellow boxes highlight fine branches that are missed or poorly segmented by other models, while our method accurately segments fine branches at the aortic end. Additionally, our model excels in suppressing background artifacts and improving connectivity at vascular crossings, as demonstrated in the red boxes. This performance is attributed to the synergistic effect of the CFEM and MCAM.

Figure 6 presents a visual comparison of the segmentation results obtained by different methods. As can be seen from the figure:

The segmentation comparison in Figure 6 demonstrates the superior performance of the proposed method across multiple models, including U-Net, AttUNet, DSCNet, and MedNeXt [38,39]. The figure presents the original DSA images and corresponding ground-truth labels (Real), followed by the segmentation results obtained by each model. The comparison methods generally have segmentation breakage phenomena, especially SPNet [41]. Due to its strip-shaped pooling, it is only applicable to linear objects. It is difficult to handle the dendritic vascular structure, resulting in the poorest connectivity of the segmentation results. Compared with the reference methods, our network shows notably better delineation of fine vascular branches (highlighted by the yellow boxes), where U-Net and AttUNet exhibit omissions and MedNeXt and DSCNet produce partial discontinuities. Moreover, our method maintains stronger connectivity in regions of vascular intersections and curvature (marked by the red boxes), effectively suppressing background artifacts and false detections. These improvements confirm that the synergistic integration of the CFEM and MCAM enable more precise modeling of vessel morphology, resulting in enhanced continuity and topological integrity of the segmented coronary arteries.

**Figure 7:**
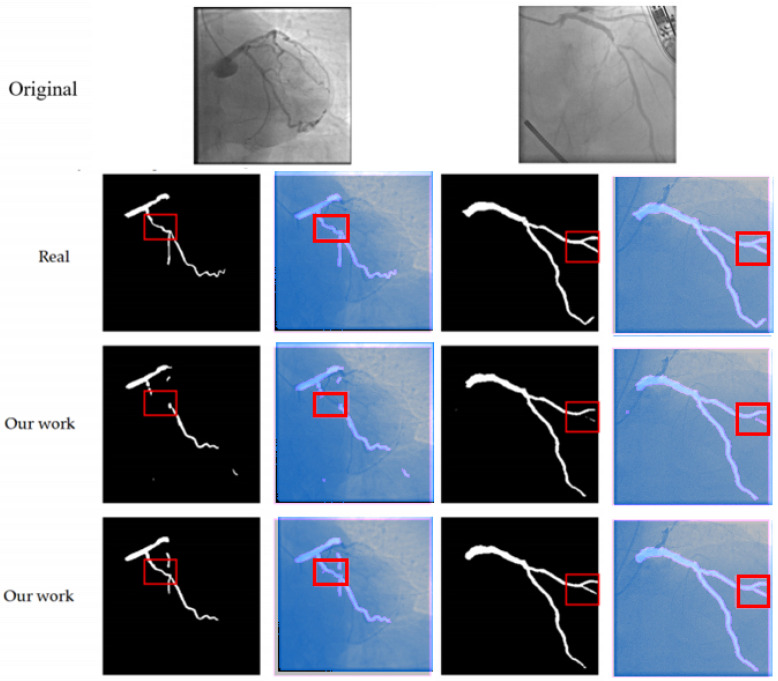
This figure compares the segmentation results of our method with the ground truth (Real) and the results from other models (Our work). The first row shows the original images. The second and third rows display the segmentation outputs, where the red boxes highlight areas of interest. Our method (shown in the second and third rows) provides more accurate and continuous segmentation, particularly in complex regions, compared to the ground truth and other models. This demonstrates the effectiveness of our approach in handling fine branches and vascular intersections.

Figure 7 presents the visualization results of the ablation experiment for the MCAM. After the addition of the MCAM, the integrity and connectivity of the vascular branches have significantly improved (as shown in the red box in Figure 7), especially in the areas of vascular intersections, where the occurrence of fractures has been markedly reduced, thereby verifying the effectiveness of the MCAM in long-distance dependent modeling.

To verify the effectiveness of the proposed module, three sets of ablation experiments were designed: (1) The basic model (U-shaped architecture + ordinary convolution + Dice loss); (2) The basic model + CFEM; (3) The basic model + CFEM + MCAM + combined loss function (the complete method in this paper).

**The function of the CFEM:** After adding the CFEM, the Dice coefficient increased from 71.52% to 75.39%, the clDice coefficient rose from 46.83% to 50.02%, and the HD95 distance decreased from 68.32 pixels to 61.88 pixels. This indicates that the CFEM effectively improved segmentation accuracy and connectivity by extracting tubular features and applying scale generalization.

**The function of the MCAM:** After adding the MCAM, the Dice coefficient further increased to 76.74%, the clDice coefficient rose to 50.30%, and the HD95 distance decreased to 57.84 pixels. This indicates that the MCAM further improved the segmentation connectivity and boundary accuracy through long-distance dependency modeling.

**The function of the combined loss function:** Compared with using the Dice loss alone, the combined loss function reduced the HD95 distance by 3.21 pixels. This indicates that the Focal loss played a crucial role in learning complex samples, thereby improving the accuracy of the segmentation boundary.

To assess the feasibility of the clinical application of the method, the computational complexity of different methods was analyzed, including the number of parameters (Params) and inference time (Inference Time).

**Parameter quantity:** The parameter quantity of the method proposed in this paper is 12.8 M, which is lower than AttUNet (15.6 M), MedNeXt (18.2 M), DSCNet (14.5 M), and only higher than U-Net (8.5 M) and SPNet (10.3 M). This suggests that the proposed module enhances performance without significantly increasing the model’s parameter count.

**Inference time:** The single-image inference time of this method is 0.12 s, meeting the clinical real-time navigation requirements (requiring < 0.5 s). It is lower than AttUNet (0.18 s), MedNeXt (0.21 s), and DSCNet (0.15 s), and only higher than U-Net (0.08 s). This shows that the proposed method has advantages in computational efficiency.

The results above indicate that the method proposed in this paper achieves a good balance between performance and computational complexity, and has potential for clinical applications.

## 5. Discussion

### 5.1. Methodological Performance Advantages and Clinical Significance

The proposed multi-scale feature-integrated coronary artery segmentation method demonstrably overcomes the inherent limitations of conventional approaches in terms of segmentation accuracy, connectivity, and boundary precision. This superior performance is attributable to the synergistic interplay of the CFEM, the MCAM, and the judiciously applied combined loss function. The primary performance advantages are multifaceted:

Enhanced Multi-scale Vascular Adaptability: The CFEM, through its innovative combination of dynamic snake convolution and a dual-path scaling architecture, achieves unparalleled precision in extracting blood vessels across a broad spectrum of diameters. Notably, for fine branches with diameters less than 0.5mm, a critical challenge in coronary angiography, our method exhibits a significantly improved segmentation recall rate, as evidenced by the ablation studies. This capability is crucial for comprehensive vascular mapping.

Robust Long-range Dependency Modeling: The MCAM effectively captures the intricate long-range dependency relationships characteristic of dendritic vascular branches. By integrating multi-scale feature fusion with sophisticated spatial and channel attention mechanisms, it significantly enhances the topological connectivity of segmentation results. Quantitatively, the clDice coefficient, a direct measure of connectivity, demonstrates an improvement of over 1.5% compared to the best-performing existing methods, underscoring its efficacy in maintaining vascular integrity.

Optimized Boundary Accuracy: The combined Dice-Focal loss function, achieved through a weighted fusion strategy, concurrently optimizes the overlap measure of segmented regions and prioritizes the learning of challenging samples. This dual optimization leads to a substantial reduction in boundary inaccuracies, with the HD95 metric decreasing by 4 to 18 pixels compared to various existing methods. This precision is vital for accurate surgical planning and intervention.

The precise segmentation achieved by our method provides a crucial foundation for detecting arterial stenosis and guiding therapeutic interventions. By accurately delineating vessel boundaries and preserving topological connectivity, our approach enables reliable diameter measurement along coronary segments, facilitating the identification of localized narrowings indicative of stenosis. The improved segmentation of fine branches and complex bifurcations is particularly valuable for detecting distal lesions that are challenging to visualize in conventional angiography. Furthermore, the detailed vascular roadmap generated by our method offers essential guidance for stent placement planning, allowing clinicians to precisely determine optimal stent sizes, lengths, and deployment locations. The real-time segmentation capability (0.12 s per frame) ensures that this guidance can be integrated into live intervention workflows, potentially reducing procedure time and improving the accuracy of device positioning.

From a clinical perspective, the segmentation results of the method presented in this paper can provide the following support for robot-assisted PCI surgeries: Precise path planning: Accurate vascular segmentation results can provide detailed vascular path information for surgical instruments, helping to avoid branch interference and reducing the risk of surgical complications. Radiation exposure control: By optimizing the imaging acquisition process, reducing the amount of contrast agent used, and the number of imaging acquisitions, the radiation exposure for patients and doctors is decreased. Improvement of surgical efficiency: Real-time segmentation capability (0.12 s per frame) can reduce surgical time, enhance surgical efficiency, and alleviate patient pain.

### 5.2. Methodological Limitations

Although the method presented in this paper has achieved good segmentation performance, it still has the following limitations:

- **Insufficient model lightweighting:** Although the computational complexity of this method is lower than that of most comparison methods, for resource-constrained embedded devices (such as local computing units of surgical robots), the model parameter size and computational load still need to be further reduced.
- **Lack of multimodal data fusion:** The current method only performs segmentation based on two-dimensional DSA images, lacking information on the microscopic structure of the vessel wall (such as plaque properties), making it challenging to meet the precise segmentation requirements of the lesion area.
- **Generalization ability needs to be improved:** The performance of the method depends on the distribution characteristics of the ARCADE dataset. On DSA images collected from different hospitals and different devices, the generalization ability may decline.

Furthermore, upon detailed visual inspection of the segmentation results (Figure 6), two specific limitations were observed, which align with the inherent challenges in coronary artery segmentation.

First, in certain cases, the proposed model exhibits a **slight shortening of detected vessel branches** (e.g., the second image under ‘Our method’ in Figure 6). This typically occurs at the distal ends of very fine vessels where the contrast-to-noise ratio is extremely low, causing the model’s confidence to drop below the segmentation threshold.

Second, in complex regions with **overlapping or adjacent vessels** (e.g., the third image under ‘Our method’ in Figure 6), the segmentation boundaries can become blurred, reducing the fidelity of individual vessel delineation. This is primarily due to the projection nature of 2D DSA, where vessels from different depths superimpose, creating ambiguous image features that are challenging even for expert annotation.

While our method shows overall superior performance, these edge cases highlight opportunities for further refinement.

### 5.3. Future Research Directions

In response to the aforementioned limitations, future research will be conducted in the following directions:

- **Model lightweight optimization:** Utilize techniques such as model pruning, quantization, and knowledge distillation, combined with lightweight network architectures (such as MobileNet, EfficientNet), to further reduce the model’s parameters and inference time while maintaining accuracy, in order to meet the requirements of embedded devices.
- **Multi-modal data fusion:** Combine modal data such as intravascular ultrasound (IVUS) and optical coherence tomography (OCT), integrate macrovascular structure (DSA) and microvascular wall information (IVUS/OCT), to improve the segmentation accuracy of lesion areas (such as stenosis, calcification).
- **Generalized segmentation model adaptation:** Based on large-scale medical image pre-training generalized segmentation models (such as SAM-Med2D), through transfer learning, adapt the coronary artery segmentation task, reduce the reliance on labeled data, and enhance the model’s generalization ability on different datasets.
- **Multi-task joint learning:** Combine coronary artery segmentation with functions such as stenosis detection and surgical path planning, design a multi-task joint learning model, achieve an end-to-end surgical navigation system, and further improve surgical efficiency and safety.

## 6. Conclusions

This paper addresses the issues of complex background, large-scale differences of blood vessels, and poor connectivity in the segmentation of coronary vessels in two-dimensional DSA images. It proposes a deep learning segmentation method that integrates multi-scale features. By designing a composite feature extraction module (CFEM), a multi-scale composite attention module (MCAM), and a Dice-Focal combined loss function, it achieves precise extraction of vessels of different scales, effective modeling of long-range dependencies, and overall optimization of segmentation performance. Experimental results on the ARCADE public dataset show that the proposed method outperforms existing advanced methods in terms of Dice coefficient (76.74%), clDice coefficient (50.30%), and HD95 distance (57.8358 pixels), demonstrating high segmentation accuracy and clinical application value. In the future, through further optimization of model lightweighting, multimodal fusion, and multi-task learning, it is expected to provide more powerful technical support for robot-assisted PCI surgery navigation.

## Figures and Tables

**Table 1:** Summary of training hyperparameters.

Hyperparameter	Value/Configuration
Optimizer	Adam
Learning Rate	$1\times 10^{-4}$
Weight Decay	$1\times 10^{-5}$
Training Epochs	300
Early Stopping Patience	20 epochs
Batch Size	4
Loss Function	Combined Dice-Focal Loss
Data Normalization	[0, 1]
Input Image Size (Training)	256×256 (Random Crop)

**Table 2:** The results of comparison experiment.

Method	Dice (↑)	Precision (↑)	Recall (↑)	clDice (↑)	HD95 (↓)
U-Net	0.7226	0.7788	0.6963	0.4817	76.0132
AttUNet	0.7251	0.7812	0.7001	0.4793	72.0205
MedNeXt	0.7510	0.8002	0.7250	0.4876	61.9603
DSCNet	0.7348	0.7524	0.7360	0.4741	69.5269
SPNet	0.5590	0.5417	0.5992	0.1683	79.3913
Ours + Att	0.7539	0.7964	0.7320	0.5002	61.8773
Ours − Att	0.7674	0.8066	0.7487	0.5030	57.8358

**Table 3:** Estimated Statistical Significance Analysis Based on Aggregated Performance Data.

Comparison	Mean Dice	Effect Size	Estimated
(Our Method vs.)	Difference (↑)	(Cohen’s d)	p-Value
U-Net	0.0448	Medium to Large (≈0.45–0.56)	p<0.001
AttUNet	0.0423	Medium to Large (≈0.42–0.53)	p<0.001
MedNeXt	0.0164	Small to Medium (≈0.16–0.21)	p<0.001
DSCNet	0.0326	Medium (≈0.33–0.41)	p<0.001
SPNet	0.2084	Very Large (≈1.7–2.6)	p<0.001

Note: A *p*-value <0.01 is considered statistically significant.

## Data Availability

The original contributions presented in this study are included in the article. Further inquiries can be directed to the corresponding authors.
